# Comparison of Cancer Mortality and Incidence Between New Zealand and Australia and Reflection on Differences in Cancer Care: An Ecological Cross-Sectional Study of 2014-2018

**DOI:** 10.1177/10732748231152330

**Published:** 2023-05-07

**Authors:** Phyu Sin Aye, Shwe Sin Win, Sandar Tin Tin, J. Mark Elwood

**Affiliations:** 1 Department of Epidemiology and Biostatistics, 1415 University of Auckland, Auckland, New Zealand

**Keywords:** cancer mortality, cancer incidence, cancer care, comparison, New Zealand, Australia

## Abstract

**Background:**

Despite many background similarities, New Zealand showed excess cancer deaths compared to Australia in previous studies. This study extends this comparison using the most recent data of 2014-2018.

**Methods:**

This study used publicly available cancer mortality and incidence data of New Zealand Ministry of Health and Australian Institute of Health and Welfare, and resident population data of Statistics New Zealand. Australian cancer mortality and incidence rates were applied to New Zealand population, by site of cancer, year, age and sex, to estimate the expected numbers, which were compared with the New Zealand observed numbers.

**Results:**

For total cancers in 2014-2018, New Zealand had 780 excess deaths in women (17.1% of the annual total 4549; 95% confidence interval (CI) 15.8-18.4%), and 281 excess deaths in men (5.5% of the annual total 5105; 95% CI 4.3-6.7%) compared to Australia. The excess was contributed by many major cancers including colorectal, melanoma, and stomach cancer in both sexes; lung, uterine, and breast cancer in women, and prostate cancer in men. New Zealand’s total cancer incidences were lower than those expected from Australia’s in both women and men: average annual difference of 419 cases (−3.6% of the annual total 11 505; 95% CI −4.5 to −2.8%), and 1485 (−11.7% of the annual total 12 669; 95% CI -12.5 to −10.9%), respectively. Comparing time periods, the excesses in total cancer deaths in women were 15.1% in 2000-07, and 17.5% in 1996-1997; and in men 4.7% in 2000-2007 and 5.6% in 1996-1997. The differences by time period were non-significant.

**Conclusion:**

Excess mortality from all cancers combined and several common cancers in New Zealand, compared to Australia, persisted in 2014-2018, being similar to excesses in 2000-2007 and 1996-1997. It cannot be explained by differences in incidence, but may be attributable to various aspects of health systems governance and performance.

## Introduction

Cancer is a challenging health problem worldwide, being responsible for nearly one in six deaths globally in 2020.^1^ Cancer burden is remarkably high in Australia and New Zealand having the highest age-standardised rates (ASRs) of cancer diagnosis internationally: 452 and 423 per 100 000 population, respectively, and age standardised mortality rates of 83 per 100 000 population in Australia, and 99 in New Zealand.^2^

Australia and New Zealand have many similarities, including the generally similar social and economic structure and publicly provided health care systems. Their training systems for medical specialists are combined, and training for other health professionals is very similar in the two countries. Both countries have a variety of ethnic and socio-economic groups, having minority groups with worse health outcomes.^3^ Despite those similarities, previous studies^4-6^ have shown that there have been constantly higher rates of cancer deaths in the whole population of New Zealand compared with Australia since the 1990s, although in both countries cancer death rates have dropped substantially. The differences in mortality were not explained by differences in incidence, which were much smaller, but were consistent with differences in case survival.^7^ The current analysis extends this comparison of cancer mortality and incidence for a further five years, from 2014 to 2018, for all cancers combined and for the most common cancers, aiming to identify potential areas for improvement in New Zealand cancer care.

## Methods

### Data Collection

This is a cross-sectional observational study using the whole population data of New Zealand and Australia published on the Ministry of Health websites. Information on New Zealand cancer deaths and new registrations from 2014 to 2018, by diagnosis year, sex, cancer site, and five-year age group from 0-4 to 85+, was obtained from publicly available national cancer data and statistics^8^ and the Mortality Web tool^9^ of the Ministry of Health (NZ). Information on Australian cancer incidence and mortality rates was obtained from publicly available national cancer data in Australia^10^ of the Australian Institute of Health and Welfare (AIHW). Estimated New Zealand resident population data by year, five-year age group and sex were taken from the Statistics New Zealand website.^11^

Cancer cases were identified using the International Classification of Diseases version 10 (ICD 10).^12^ The included ICD-10 codes were C33-34 (Lung), C18, 19, 20 (Colorectal), C25 (Pancreas), C82-86 (Non-Hodgkin Lymphoma), C43 (Melanoma), C71 (Brain), C15 (Esophagus), and C16 (Stomach) for both sexes; C61 (Prostate) for male; C50 (Breast), C56 (Ovary), C54, 55 (Uterus), and C53 (Cervix) for female.

### Statistical Analysis

For each sex, year and cancer, the Australian incidence and mortality rates for each 5-year age group were applied to New Zealand population data to obtain expected, E, numbers of cancers, to be compared to the actual observed, O, numbers (indirect standardisation).^13^ Results are expressed as the O-E difference as a percentage of the observed New Zealand numbers, with their 95% confidence limits. Data analyses were performed using Stata v16 and Microsoft Excel. The reporting of this study conforms to STROBE guidelines.^14^

### Ethics Considerations

This study used non-identifiable aggregated publicly available data; therefore, it did not require ethics approval.

## Results

### Cancer Mortality

Over the five-year period from 2014 to 2018, New Zealand had on average each year 9654 deaths from cancer, 4549 in women and 5105 in men (Table 1). Applying the Australian death rates to the New Zealand population showed that on average each year there had been 780 excess deaths in women (17.1% of the annual total, 95% confidence interval (CI) 15.8 to 18.4%), and 281 excess deaths in men (5.5% of the annual total, 95% CI 4.3 to 6.7%).

**Table 1.** Annual Average Deaths From Cancers in New Zealand, by Sex and Cancer Site, in Comparison with Australia, Showing 2014-2018, 2000-2007, and 1996-1997.

	2014-2018				2000-2007^b^				1996-1997^c^			
	NZ annual deaths	Excess or deficit	% Excess or deficit (95% CI)		NZ annual deaths	Excess or deficit	% Excess or deficit(95% CI)		NZ annual deaths	Excess or deficit	% Excess or deficit (95% CI)	
Both sexes	9654	1060	11.0	(10.1 to 11.9)	7998	765	9.6	(7.4 to 11.8)	7372	832	11.3	(9.7 to 12.9)
Female												
All cancers	4549	780	17.1	(15.8 to 18.4)	3771	568	15.1	(11.9 to 18.3)	3519	616	17.5	(15.2 to 19.9)
Lung	851	196	23.1	(20.1 to 26.1)	612	120	19.7	(11.9 to 27.9)	516	119	23.0	(17.0 to 29.3)
Colorectal & anus	678	223	32.9	(29.6 to 36.3)	586	204	34.9	(27.0 to 43.4)	530	115	21.7	(15.8 to 27.9)
Breast	660	102	15.5	(12.1 to 19.0)	632	120	18.9	(11.3 to 27.0)	651	143	22.0	(16.7 to 27.6)
Pancreas	260	1	0.3	(−5.1 to 5.9)	171	−11	−6.4	(−20.9 to 9.7)	156	−5	−3.2	(−14.0 to 8.5)
Ovary	186	3	1.5	(−4.8 to 8.1)	194	34	17.7	(4.1 to 32.8)	173	23	13.3	(3.0 to 24.4)
NHL	135	16	11.9	(4.5 to 19.7)	135	6	4.3	(−11.8 to 22.7)	144	7	4.6	(−6.7 to 16.8)
Uterus	132	35	26.8	(19.3 to 34.7)	88	26	30.0	(10.2 to 53.4)	64	11	16.8	(.1 to 35.8)
Melanoma	119	30	25.1	(17.2 to 33.5)	103	27	26.8	(8.3 to 48.1)	84	20	23.7	(9.1 to 40.1)
Brain	112	4	3.6	(−4.5 to 12.2)	90	1	1.5	(−18.1 to 24.5)	—			
Stomach	105	26	24.8	(16.5 to 33.8)	113	29	25.7	(8.1 to 46.0)	—			
Esophagus	75	11	14.3	(4.5 to 25.0)	72	9	12.5	(−9.2 to 38.5)	—			
Cervix	52	6	12.4	(.6 to 25.4)	62	16	26.4	(3.0 to 54.7)	78	20	25.4	(10.2 to 42.4)
All others^a^	1183	126	10.6	(8.1 to 13.2)	914	−15	−1.7	(−8.0 to 5.0)	—			
Male												
All cancers	5105	281	5.5	(4.3 to 6.7)	4227	197	4.7	(1.7 to 7.7)	3853	215	5.6	(3.4 to 7.8)
Lung	911	−26	−2.8	(−5.7 to .1)	859	−17	−2.0	(−8.6 to 4.9)	893	4	0.4	(−4.2 to 5.2)
Colorectal & anus	722	187	25.9	(22.7 to 29.2)	589	143	24.3	(16.4 to 32.7)	581	104	17.9	(12.3 to 23.9)
Prostate	657	74	11.2	(7.9 to 14.7)	577	54	9.4	(1.4 to 17.9)	514	30	5.8	(−.2 to 12.1)
Pancreas	257	−22	−8.7	(−14.1 to −3.1)	166	−21	−12.7	(−27.3 to 3.8)	—			
Melanoma	226	51	22.7	(16.9 to 28.7)	162	19	11.5	(−3.3 to 28.2)	114	4	3.2	(−9.3 to 17.1)
NHL	180	15	8.5	(2.1 to 15.3)	159	11	7.1	(−7.9 to 23.9)	145	0	−0.2	(−11.4 to 12.0)
Stomach	177	43	24.2	(17.7 to 31.0)	181	46	25.2	(11.2 to 40.9)	166	22	13.0	(2.5 to 24.4)
Esophagus	167	−12	−6.9	(−13.6 to .1)	133	−8	−6.0	(−22.3 to 12.5)	—			
Brain	157	−6	−3.6	(−10.4 to 3.7)	134	12	9.0	(-7.2 to 27.5)	116	3	2.3	(−10.2 to 16.0)
All others^a^	1652	−24	−1.5	(−3.6 to .7)	1268	−42	−3.3	(−8.8 to 2.3)	—			
All except prostate	4448	207	4.7	(3.3 to 6.0)	3650	143	3.9	(.7 to 7.2)	—			

^a^All cancers minus the cancers above.

^b^Alafeishat et al. (2014).

^c^Skegg et al. (2002).

The proportional differences were greatest for cancers of the colorectum, being seen in both sexes (33% excess in women, 26% in men). Statistically significant excesses were seen for melanoma, stomach cancer, and non-Hodgkin lymphoma in both sexes; for cancers of the lung, uterus, breast, esophagus, and cervix in women, and for prostate cancer in men. Thus for lung cancer, there was a substantial 23% excess in women, but no excess in men.

In comparison, in 1996-1997 there were 17.5% more cancer deaths in women in New Zealand than in Australia, adjusted for type of cancer and age, and 5.6% more deaths in men (Table 1), In 2000-2007 these differences showed slight reductions to 15.1% in women and 4.7% in men. In the 2014-2018 period, the excesses have increased slightly, to be similar to those in 1996-1997. These differences between time periods were non-significant.

The pattern of excesses by cancer site was generally similar in the three time periods. Although non-significant, the substantial excesses in cancers of the colorectum and stomach in both sexes were similar in the later 2 periods, but greater than in the earliest period. In women, however, the excess deaths in non-Hodgkin lymphoma (NHL) almost tripled (11.9% in 2014-2018, compared to 4.3% and 4.6% in the earlier periods) while those of the lung, uterus, and esophagus changed only slightly. The excesses in breast cancer deaths decreased slightly over time, to 15.5% in 2014-2018 from 22% in 1996-1997. In men, the excess percent of melanoma increased over time (22.7% in 2014-2018), although based on small numbers. None of these site-specific changes were statistically significant.

### Cancer Incidence

The equivalent analysis for cancer incidence for 2014-2018 and 2000-2007 shows that these mortality excesses in New Zealand were not produced by equivalent excesses in incidence (Table 2). Cancer incidence data was not assessed in the 1996-1997 study. Indeed, New Zealand’s total cancer incidences were lower than those expected from Australian rates in both women, average annual difference of 419 cases (−3.6% of the annual total of 11 505, 95% CI -4.5 to −2.8%) and men, average annual difference being 1485 (−11.7% of the annual incidence total of 12 669, 95% CI −12.5 to −10.9%). The incidence rates of stomach cancer, melanoma, prostate cancer, and breast cancer were decreased in New Zealand, despite the higher death rates. Colorectal cancer incidence was significantly higher in New Zealand by 10% in women and 4% in men, which was less than the mortality excess.

**Table 2.** Annual Average Incidence Cases of Cancers in New Zealand, by Sex and Cancer Site, in Comparison With Australia, 2014-2018.

	2014-2018				2000-2007^b^			
	NZ annual cases	Excess or deficit	% Excess or deficit(95% CI)		NZ annual cases	Excess or deficit	% Excess or deficit(95% CI)	
Both sexes	24 174	−1904.61	−7.9	(−8.4 to −7.3)	18 576	−178	−1	(−2.4 to .5)
Female								
All cancers	11 505	−419	−3.6	(−4.5 to −2.8)	8782	287	3.3	(1.2 to 5.4)
Breast	3341	−87	−2.6	(−4.1 to −1.1)	2403	25	1.0	(−2.9 to 5.1)
Colorectal	1478	141	9.5	(7.3 to 11.8)	1324	−338	−25.5	(−30.9 to −20.0)
Melanoma	1117	−32	−2.9	(−5.5 to −.2)	922	96	10.4	(4.0 to 17.1)
Lung	1103	80	7.2	(4.6 to 9.9)	722	93	12.9	(5.8 to 20.5)
Uterus	572	30	5.3	(1.7 to 9.0)	341	3	0.8	(−9.5 to 12.0)
NHL	392	−57	−14.6	(−18.9 to −10.0)	301	−31	−10.3	(−21.3 to 1.7)
Pancreas	289	−30	−10.5	(−15.6 to −5.2)	183	−15	−8.2	(−22.2 to 7.4)
Ovary	259	53	20.4	(15.1 to 26.0)	296	59	20.1	(9.0 to 32.1)
Cervix	163	−4	−2.2	(−9.0 to 4.9)	172	26	15.1	(.8 to 31.2)
Stomach	142	−7	−4.7	(−11.9 to 3.0)	143	12	8.5	(−7.2 to 26.4)
Brain	134	−5	−3.6	(−11.1 to 4.3)	108	−8	−7.4	(−-25.5 to 13.4)
Esophagus	87	7	8.1	(−1.1 to 17.9)	83	11	13.2	(−7.2 to 37.2)
All other^a^	2427	−508	−20.9	(−22.7 to −19.1)	1784	354	19.8	(15.2 to 24.6)
Male								
All cancers	12 669	−1485	−11.7	(−12.5 to −10.9)	9794	−465	−4.7	(−6.7 to −2.7)
Prostate	3521	−185	−5.3	(−6.7 to −3.8)	2756	−2	−0.1	(−3.8 to 3.7)
Colorectal	1649	71	4.3	(2.2 to 6.5)	1363	−7	−0.5	(−5.8 to 4.9)
Melanoma	1399	−196	−14.0	(−16.4 to −11.7)	978	−88	−9.0	(−15.2 to −2.5)
Lung	1144	−168	−14.6	(−17.2 to −12.0)	972	−102	−10.5	(−16.7 to −4.0)
NHL	495	−92	−18.7	(−22.6 to −14.6)	347	−49	−14.1	(−24.4 to −3.0)
Pancreas	298	−48	−16.2	(−21.2 to −11.0)	177	−27	−15.2	(−29.4 to .6)
Stomach	257	−22	−8.5	(−13.9 to −2.9)	230	−3	−1.3	(−13.8 to 12.5)
Esophagus	198	−9	−4.8	(−10.9 to 1.6)	161	−13	−8.1	(−23.0 to 8.6)
Brain	191	−14	−7.3	(−13.6 to −.8)	152	−3	−2.0	(−17.2 to 15.2)
All other^a^	3515	−821	−23.4	(−24.8 to −21.9)	2659	−171	−6.4	(−10.2 to −2.6)
All except prostate	9148	−1300	−14.2	(−15.1 to −13.3)	7038	−463	−6.6	(−8.9 to −4.2)

^a^All cancers minus the cancers above.

^b^Alafeishat et al (2014).

### Trends in Total Cancer Mortality From 1990 to 2018

**Figure 1.**
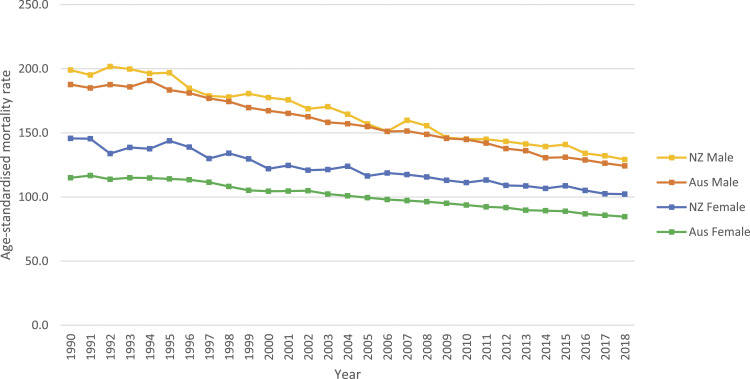
Age-standardised mortality rates of all cancers, by sex and year, New Zealand and Australia, 1990-2018.

Over the 28 year period from 1990 to 2018 (Figure 1), age standardised mortality rates for total cancer had declined substantially in both New Zealand and Australia, but the excess death rate in New Zealand had persisted throughout that period.

## Discussion

### Mortality Differences

In this study we have compared deaths from cancer between the entire populations of New Zealand and Australia, adjusting for population numbers, sex and age structure of the population, and case-mix (types of cancer). For both sexes, in 2014 to 2018, the most recent years available, there were 11.0% excess deaths in New Zealand, 1061 deaths per year. These are deaths which would not have occurred had the sex, age, and cancer type-specific death rates recorded in Australia also applied to New Zealand. This excess has persisted over time; previous studies using identical methods showed 9.6% excess in New Zealand in 2000-2007,^5^ and 11.3% excess in 1996-1997.^4^ The excesses have been larger for women – 17.1% in the current study, compared to 15.1% in 2000-2007 and 17.5% in 1996-1997. For men, the equivalent excesses have been 5.5%, 4.7%, and 5.6%, respectively.

### Relationship to Incidence and Survival

The excesses in cancer deaths cannot be explained by a higher cancer incidence in New Zealand. In the current study, there were 7.9% fewer incident cancer cases per year in New Zealand. For 2000-2007, there were 1% fewer incident cancers in New Zealand.^5^ The earlier study for 1996-1997 applied a different method to the incidence calculations, and showed a 1% higher incidence rate in New Zealand. Incidence rates may be affected by overdiagnosis, which has been estimated in Australia^15^; yet, comparable data is unavailable for New Zealand.

The higher mortality in New Zealand is consistent with lower survival, with 5-year relative survival ratios for all cancers diagnosed in 2006-2010 being 4.2% lower in women, and 3.8% lower in men in New Zealand, compared to Australia.^7^ It is also consistent with an analysis restricted to cancer deaths within five years of diagnosis.^6^

### Quality of Data

It is unlikely that these excesses could be produced by differences between the countries in the recognition and recording of cancer deaths. Both countries have long established mortality recording systems following international procedural and coding practices. Cancer incidence recording is open to more variations in practice than is mortality, but both countries have legally protected nationally coordinated cancer registries following international best practice, and are included in international analyses with extensive quality control.^16^

### Variations in Site-specific Cancers

#### Colorectal cancer

In this analysis, New Zealand has much higher mortality in comparison to Australia (33% excess in women, 26% in men), but differences in the incidence rates were less pronounced (9.5% excess in women, 4% in men). In the previous period 2000-07 there were similar excesses in mortality, but no excess in incidence. This may partially be explained by the earlier implementation of the colorectal cancer screening program in Australia (2006) in comparison to New Zealand (2017). In the earliest period, 1996-97, the excesses were slightly smaller (22% in men, 18% in women). A recent International Cancer Benchmarking Partnership (ICBP) study^17^ found that of the 7 countries studied, Australia had the lowest proportion of patients diagnosed with distant stage (21.5% for colon cancer and 17.7% for rectal cancer) but New Zealand had the highest (28.2% for colon cancer and 25.8% for rectal cancer); 1- and 5-year survival was comparable between the two countries in patients with localised or regional cancer but was poorer in New Zealand patients with distant stage, indicating that there may also be differences in management of metastatic cancer between the two countries.

#### Lung cancer

We found higher mortality in New Zealand women (23% excess) in comparison to Australian women although the difference in the incidence rates was less apparent (7% excess). The mortality rates in men were similar between the two countries but New Zealand men had a lower incidence. A recent ICBP study^18^ found that a larger proportion of New Zealand patients was diagnosed at later stages compared to other countries including Australia; women generally were diagnosed at an earlier stage and had more favourable outcomes.

An earlier study reported that New Zealand lung cancer patients had a lower rate of initial anti-cancer treatment compared to Australia and the US, which had contributed to poorer survival outcomes.^19^ Another study reported that late diagnosis, low rates of curative treatment for non-metastatic disease and long transit times from diagnosis to treatment partially explained ethnic differences in lung cancer survival within New Zealand.^20^

#### Melanoma

This analysis shows higher mortality rates in New Zealand men and women compared to Australia (23% excess in men, 25% in women), but the incidence rates in New Zealand were lower in men, and similar in women, to incidence rates in Australia in the 2014-18 period. The mortality difference between New Zealand and Australia could be due to differences in the extent of disease at diagnosis or in the distribution of melanoma subtypes.^21,22^ The differences seem inconsistent with the similarity in reported survival ratios in the two countries.^7,16^ While neither country has a screening programme for melanoma, screening is extensively done in primary care and private facilities in both countries, with little quality control.^23^

#### Prostate cancer

New Zealand men had higher mortality but lower incidence compared to their Australian counterparts. The findings are difficult to interpret due to wide variations in the incidence and survival of prostate cancer since the introduction of PSA in the cancer diagnostic and screening processes. In neither country is there a formal screening programme; primary care physicians usually provide opportunistic screening to asymptomatic men.

#### Breast cancer

New Zealand women had higher mortality compared to their Australian counterparts (16% excess) although the incidence in the two countries was similar. Both countries have national screening programmes offering two-yearly mammography; in 2016 the reported participation rate in 2016 was higher in New Zealand (71%) compared to 54% in Australia.^24,25^ The excess deaths in New Zealand have decreased slightly over time, having been 19% in 2000-07 and 22% in 1996-97.

Our earlier studies showed that survival from breast cancer in Australia was only slightly higher at one year, but the differences were more pronounced at five and 10 years.^7,26^ This suggests that diagnosis may be comparable in the two countries but differences in further treatment may be important. The relationship between the increased use of adjuvant hormonal and chemotherapy and reductions in breast cancer mortality in Victoria, Australia^27^ shows that the extent of systemic therapy use and the timing of its introduction are important.

### Factors Influencing Cancer Mortality

Why is there such a substantial difference in cancer deaths between two generally similar countries? The reasons may be all those influencing the effectiveness of a health system, and can be described in terms of six key aspects of health systems identified by WHO and others.^28^

#### Governance and leadership

A conceptual approach to cancer control in New Zealand began during the 1990s focused on cervical, skin, breast and lung cancers, but not linked to health programme changes.^29^ A New Zealand Cancer Control Strategy in 2003 set goals for improving access to high quality and timely cancer services, emphasising disparities in cancer outcomes between subgroups.^30^ Regional Cancer Networks were set up in 2006–08, setting service standards for 10 major cancers,^31^ but without methods to ensure compliance.^32^ In December 2019, Te Aho o Te Kahu, the Cancer Control Agency was set up, reporting direct to the Minister of Health, with a full time director (https://teaho.govt.nz), and produced a major review of cancer.^33^

In Australia, Cancer Australia was set up in 2006 (https://www.canceraustralia.gov.au), by the federal government, in association with voluntary sectors and state governments. It was preceded by the National Cancer Control Initiative from 1997.^34^ Cancer Australia does not provide services, but makes recommendations to the Australian federal government and other groups about cancer policy and priorities. The agency also focuses on populations who experience poorer health outcomes, including indigenous peoples and those in rural and remote areas.

Clinical practice guidelines for most common cancers have been developed in both countries, with some being jointly developed. In neither country has there been a systematic system of monitoring clinical practice for comparison to the guidelines. In Australia, there have been many national or state systematic surveys of the management of cancer, which have identified variations in management and gaps between optimal management and actual practice.^35^ In New Zealand, there have been few national studies of cancer management, apart from a national study of colorectal cancer,^36^ but many localised or site specific studies.

In an ICBP study involving key informants in 13 jurisdictions including Victoria, Western Australia and New Zealand in 2019-20, it was concluded that improving cancer outcomes requires effective political and clinical leadership.^37^ Appointing a central agency, involving clinicians, and ensuring strong clinical leadership with a consistent political mandate were emphasised. New Zealand informants commented that the New Zealand 2003 cancer control strategy and 2005-10 cancer plans were good plans but produced little change because of insufficient commitment and leadership, and the fragmentation of services between 20 district health authorities inhibited progress.

#### Health systems financing

At the macro-economic level, cancer survival is generally better in countries which are stronger economically.^16,38^ For European countries, a linear positive trend between cancer survival and total national expenditure on health (TNEH), expressed as purchasing power parity, with an R^2^ of .8, has been shown^39^; and most studies within a country show an association between spending and better outcomes.^38^ However, within areas in the United States there was not consistent evidence, with some studies reporting increased spending as associated with worse outcomes, or no association.^38,40^ This suggests that at high levels of expenditure further increments may not be beneficial and may indicate wasteful spending.^40^ Comparing many countries using 2012 data, a positive relationship between total health expenditure per capita and a measure of overall relative cancer survival was shown.^41^ Of 30 countries with survival ratios over .55, Australia was ranked second in survival and fifth in expenditure; New Zealand was 22nd in survival and 15th in health expenditure. Australia had higher survival by 13%, consistent with the current analysis, but had 46% higher expenditure. The excess death rate for cancer in New Zealand compared to Australia is greater than for all other causes of death (9.8% compared with 7.0%, based on age-standardised rates for 2016).^42^

Data on resources specifically for cancer care are more limited. A study comparing cancer care expenditures per incident patient with ‘amenable’ cancer mortality compared 16 OECD countries, including Australia but not New Zealand.^43^ ‘Amenable’ cancer mortality was defined as deaths which could be expected to be avoidable by optimum care. Lower mortality was associated with higher spending, and greater decreases in mortality over time were seen in countries with greater increases in spending.

#### Health service delivery

Many aspects of health systems could contribute to mortality differences, such as the coordination of hospital and community care especially at the diagnostic, post primary treatment, and end of life phases.^44^ Based on a literature review and a stakeholder survey within the ICBP network, a dynamic conceptual model depicting causal pathways which may influence survival at different stages of the patient journey has been developed.^45^ This shows the complexity of health system components and their interactions, and highlights potential ‘breakpoints’ where lack of coordination may have adverse effects.

In both countries, the usual initial process apart from screening programs is that patients recognise that something is wrong, and seek help from a primary care practitioner, usually a medical general practitioner (GP). For this process to start, the patient or their family or friends has to recognise that something may be wrong. Thus knowledge, beliefs and attitudes to health states and possible abnormalities, health literacy, will be important.^46^ The ICBP assessed cancer awareness and beliefs in Canada, Europe and Australia (again Victoria and New South Wales)^47^; barriers to symptomatic presentation were reported as lower in Australia than in the UK, but there was no association with 1-year survival rates. New Zealand was not included in that survey.

The GP then is responsible for organising appropriate diagnostic tests, and when there is a substantial suspicion of cancer, referring the patient to a specialist or hospital. The process from first recognition of an issue by the patient to confirmation of the diagnosis can be complex and lengthy. Improving this process is seen as a major contributor to improvements in cancer survival, and has been given a high priority, for example in the UK and Denmark.^48^ Systems to allow GPs to order or perform tests directly, rather than having to involve a specialist, have been encouraged in Australia; in contrast, respondents in New Zealand have commented on the lack of direct access to diagnostics by GPs, and lack of diagnostic resources.^48^

An ICBP study in New Zealand primary care compared results with two Australian states, Victoria and New South Wales, and to 11 other jurisdictions.^49^ This showed that GPs’ access to tests and to specialist advice was more limited and wait times for testing were longer in New Zealand; for example, 41% of New Zealand GPs reported that they could get a referral for a suspected cancer patient within 48 hrs, compared to 60% and 59% in the Australian regions; average times for a colonoscopy were 20 weeks in New Zealand, compared to 5-6 weeks in Australia.

Cancer first presenting to hospital as or soon after an emergency admission has a reduced survival rate, and may indicate failure of the usual referral processes.^50^ In an ICBP study of eight selected types of cancers diagnosed in 2012-17 in 14 jurisdictions, the proportion with emergency presentation was highest in New Zealand, while Australian areas were similar to most other countries.^51^

Once referred to hospital, waiting times for a first specialist assessment and for start of treatment are important, and as these are hospital issues they have had more attention in national plans, such as the ‘faster cancer care’ program in New Zealand.^32,52^

#### Health workforce

Information on the workforce is similarly sparse, as most cancer care is provided by those also caring for non-cancer patients. An ICBP study was based on semi-structured interviews in 2019-20 with key informants in seven jurisdictions, including Australia and New Zealand.^48^ Shortages of staff for diagnosis services – radiologists, endoscopists, and pathologists - were noted for New Zealand, as was a lack of capital funding for radiotherapy capacity. Australia is noted as having considerably increased radiotherapy capacity in recent years. Increasing unmet demand for medical oncology services was reported generally, even though Australia was noted having ‘invested heavily’ in its medical oncology workforce. A worldwide workforce survey reported 272 cancer cases per ‘clinical oncologist’ in Australia and 525 cases per oncologist in New Zealand^53^; this was based on multiple sources ranging from published data to expert opinion, and ‘oncologist’ is defined as a specialist exclusively caring for cancer patients, which will only cover a small portion of the workforce.

#### Access to cancer medicines

Both countries have national agencies, which evaluate new therapeutic agents after applications, usually from drug companies, and are also large scale drug purchasing agencies, with considerable leverage to negotiate prices. These agencies approve drugs which are then provided free or at little cost through the public system. The assessment systems and thresholds vary between Australia and New Zealand. In 2016, there were 89 cancer medicines publicly funded in both countries; a further 35 were funded only in Australia, and 13 funded only in New Zealand.^54^ Many cancer drugs are licensed for several indications; in a survey of 10 specific cancer drugs in 13 countries, New Zealand had the fewest accepted indications, and England, Scotland and Australia had the next lowest.^55^ A study of 18 countries found manufacturers’ prices in Australia and New Zealand were generally similar to those in Europe,^56^ but the survey could not assess the actual prices negotiated. The New Zealand Pharmaceutical Management Agency (Pharmac) has thus frequently been criticised in the media and by some patient advocacy groups and oncologists for its more limited approach. In some situations, such as Herceptin for breast cancer, lobbying has led to the government overriding Pharmac’s decision and funding the drug.^57^ However a careful analysis of the situation in 2016, by authors from Pharmac and an independent oncologist, concluded that most of the cancer drugs only approved in Australia did not deliver clinically meaningful health gains assessed by objective standards.^54^ Thus the availability of more cancer drugs in New Zealand may not substantially contribute to the mortality differences, although it will be important to some patients, and perhaps to quality of life even if not to survival. A recent report^58^ states that 18 targeted cancer medicines, for 20 indications, would be likely to offer substantial clinical benefit and were available in Australia but not in New Zealand.

#### Access to other technology

An Organisation for Economic Co-operation and Development (OECD) report^59^ shows data for 2010 on numbers of equipment units per million population, showing higher levels in Australia, compared to New Zealand, of radiotherapy equipment (3% higher), PET scanners (17%), and CT scanners (173%), but lower levels of mammography machines (−5%) and MRI scanners (−45%). However, the data are derived partially from questionnaires, and may vary in the definitions, in whether private facilities are included, and in other ways.

### Strengths and Limitations of the Study

The use of the whole population data in both countries and a comprehensive review on cancer care in both countries using the WHO’s six key aspects of health systems are the strengths of this study. In terms of limitations, a more detailed comparison of specific cancers, the subgroups of the population, or specific health systems aspects using individual indicators, such as the use of particular cancer medicines, screening or technologies, between the two countries may provide opportunities to recommend the areas for improvement in New Zealand cancer care more specifically in the future.

## Conclusions

Compared to Australia, excess deaths from all cancers in New Zealand, for both male and female, persisted over the current 2014-2018 study period, as previously seen in the 2000-2007 study. The proportional excess deaths in New Zealand were greatest for colorectal cancer, and significant for melanoma, stomach cancer, and non-Hodgkin lymphoma in both sexes; for cancers of the lung, uterus, breast, esophagus, and cervix in women, and for prostate cancer in men. The excess in mortality cannot be explained by differences in incidence, but may be attributable to various aspects of health systems governance and performances.

## Supplemental Material

Supplemental Material - A Comparison of Cancer Mortality and Incidence Between New Zealand and Australia and Reflection on Differences in Cancer Care: An Ecological Cross-Sectional Study of 2014-2018 Click here for additional data file. Supplemental Material for A Comparison of Cancer Mortality and Incidence Between New Zealand and Australia and Reflection on Differences in Cancer Care: An Ecological Cross-Sectional Study of 2014-2018 by Phyu Sin Aye, Shwe Sin Win, Sandar Tin Tin, and J. Mark Elwood in Cancer Control.

## References

[1] World Health Organization . Cancer. World Health Organization. Published 2020. https://www.who.int/news-room/fact-sheets/detail/cancer. Accessed June 8, 2022.

[2] International Agency for Research on Cancer . Cancer Today. GLOBOCAN; 2020 https://gco.iarc.fr/today/home. Published 2020. Accessed June 8, 2022.

[3] Phillips B Daniels J Woodward A Blakely T Taylor R Morrell S . Mortality trends in Australian Aboriginal peoples and New Zealand Maori. Popul Health Metrics. 2017;15(1):1-12. doi:10.1186/s12963-017-0140-6 PMC5496180 28680369

[4] Skegg DC McCredie MR . Comparison of cancer mortality and incidence in New Zealand and Australia. N Z Med J. 2002;115(1153):205-208. 12064704

[5] Alafeishat L Elwood M Ioannides S . Cancer mortality and incidence trends comparing New Zealand and Australia for the period 2000–2007. N Z Med J. 2014;127(1400):9-19. 25145363

[6] Sandiford P Abdel-Rahman ME Allemani C Coleman MP Gala G . How many cancer deaths could New Zealand avoid if five-year relative survival ratios were the same as in Australia? Aust N Z J Publ Health. 2015;39(2):157-161. doi:10.1111/1753-6405.12344 25716332

[7] Aye PS Elwood M Stevanovic V . Comparison of cancer survival in New Zealand and Australia, 2006–2010. N Z Med J. 2014;127(1407):14-26. 25530328

[8] Ministry of Health . Cancer Data and Stats. New cancer Regist; 2018. https://www.health.govt.nz/nz-health-statistics/health-statistics-and-data-sets/cancer-data-and-stats

[9] Ministry of Health . Mortality Web Tool. New Zealand Mortality Collection; 2019 https://minhealthnz.shinyapps.io/mortality-web-tool/. Published 2019. Accessed March 15, 2022.

[10] Australian Institute of Health and Welfare . Cancer Data in Australia. Canberra: AIHW; 2020. https://www.aihw.gov.au/reports/cancer/cancer-data-in-australia/data

[11] Statistics New Zealand . Population Estimates - DPE. 2022. https://infoshare.stats.govt.nz/default.aspx?AspxAutoDetectCookieSupport=1

[12] World Health Organization . International Classification of Diseases Version 10. World Health Organization; 2019. https://icd.who.int/browse10/2019/en. Published 2019. Accessed June 8, 2022.

[13] Altman D Machin D Bryant T Gardner M . Statistics with Confidence: Confidence Intervals and Statistical Guidelines. 2nd ed. Wiley; 2013.

[14] Von Elm E Altman DG Egger M Pocock SJ Gøtzsche PC Vandenbroucke JP . The Strengthening the Reporting of Observational Studies in Epidemiology (STROBE) statement: Guidelines for reporting observational studies. Ann Intern Med. 2007;147(8):573-577. doi:10.7326/0003-4819-147-8-200710160-00010 17938396

[15] Glasziou PP Jones MA Pathirana T Barratt AL Bell KJL . Estimating the magnitude of cancer overdiagnosis in Australia. Med J Aust. 2020;212(4):163-168. doi:10.5694/mja2.50455 31858624 PMC7065073

[16] Allemani C Matsuda T Di Carlo V , et al. Global surveillance of trends in cancer survival 2000–14 (CONCORD-3): Analysis of individual records for 37 513 025 patients diagnosed with one of 18 cancers from 322 population-based registries in 71 countries. Lancet. 2018;391(10125):1023-1075. doi:10.1016/S0140-6736(17)33326-3 29395269 PMC5879496

[17] Araghi M Arnold M Rutherford MJ , et al. Colon and rectal cancer survival in seven high-income countries 2010-2014: Variation by age and stage at diagnosis (the ICBP SURVMARK-2 project). Gut. 2021;70(1):114-126. 32482683 10.1136/gutjnl-2020-320625

[18] Araghi M Fidler-Benaoudia M Arnold M , et al. International differences in lung cancer survival by sex, histological type and stage at diagnosis: An ICBP SURVMARK-2 Study. Thorax. 2021;77:378-390. doi:10.1136/thoraxjnl-2020-216555 34282033

[19] Stevens W Stevens G Kolbe J Cox B . Lung cancer in New Zealand: Patterns of secondary care and implications for survival. J Thorac Oncol. 2007;2(6):481-493. doi:10.1097/JTO.0b013e31805fea3a 17545842

[20] Stevens W Stevens G Kolbe J Cox B . Ethnic differences in the management of lung cancer in New Zealand. J Thorac Oncol. 2008;3(3):237-244. 18317065 10.1097/JTO.0b013e3181653d08

[21] Sneyd MJ Cox B . Clinical and histologic factors associated with melanoma thickness in New Zealand Europeans, Maori, and Pacific peoples. Cancer. 2010;117(11):2489-2498. 24048797 10.1002/cncr.25795

[22] Sneyd MJ Cox B . A comparison of trends in melanoma mortality in New Zealand and Australia: The two countries with the highest melanoma incidence and mortality in the world. BMC Cancer. 2013;13(1):372. 23915380 10.1186/1471-2407-13-372 PMC3750694

[23] Elwood JM Slevin T . Skin cancer prevention and screening. In: Eeles R Berg CD Tobias J , eds. Cancer Prevention and Screening: Concepts, Principles and Controversies; 2018:275-294.

[24] Ministry of Health . BSA New Zealand District Health Board Coverage Report: Period Ending 30 June 2018. Wellington: Ministry of Health; 2018.

[25] Cancer Australia . Breast Screening Rates. 2020. https://ncci.canceraustralia.gov.au/screening/breast-screening-rates/breast-screening-rates. Accessed 22 Apr, 2022.

[26] Elwood JM Aye PS Tin ST . Increasing disadvantages in cancer survival in New Zealand compared to Australia, between 2000-05 and 2006-10. Handelsman DJ , ed. PLoS One. 2016;11(3):e0150734. doi:10.1371/journal.pone.0150734 26938056 PMC4777383

[27] Burton RC Bell RJ Thiagarajah G Stevenson C . Adjuvant therapy, not mammographic screening, accounts for most of the observed breast cancer specific mortality reductions in Australian women since the national screening program began in 1991. Breast Cancer Res Treat. 2012;131(3):949-955. 21956213 10.1007/s10549-011-1794-6

[28] World Health Organization . Monitoring the Building Blocks of Health Systems: A Handbook of Indicators and Their Measurement Strategies, Vol. 35. Geneva: WHO; 2010. http://www.annualreviews.org/doi/10.1146/annurev.ecolsys.35.021103.105711

[29] Gavin J Marshall B Cox B . Progress towards a New Zealand Cancer Control Strategy. New Zealand: New Zealand Ministry of Health; 2001.

[30] Cancer Control Taskforce . The New Zealand Cancer Control Strategy Action Plan 2005 – 2010. Wellington: Ministry of Health; 2005. http://www.cancercontrolnz.govt.nz/

[31] Ministry of Health . New Zealand Cancer Plan Better 2015–2018. Wellington: Faster Cancer Care; 2014. http://www.health.govt.nz/system/files/documents/publications/new-zealand-cancer-plan-2015-2018-dec14.pdf. Accessed March 15, 2018.

[32] Sarfati D Jackson C . Context of cancer control in New Zealand. J Cancer Policy. 2020;23:100211.

[33] Cancer Control Agency . State of Cancer in New Zealand 2020. Wellington: Te Aho o Te Kahu, Cancer Control Agency; 2021.

[34] Anderiesz C Elwood M Hill DJ . Cancer control policy in Australia. Aust N Z Health Pol. 2006;3:12. 10.1186/1743-8462-3-12 PMC1634863 17059613

[35] Staples M Elwood M John JS Howes F Pedersen K . Perceived impact on clinical practice and logistical issues in clinical management surveys of cancer: Australian experience. Qual Saf Health Care. 2009;18(3):195-198. 19468001 10.1136/qshc.2007.024398

[36] Sharples KJ Firth MJ Hinder VA , et al. The New Zealand PIPER Project: Colorectal cancer survival according to rurality, ethnicity and socioeconomic deprivation-results from a retrospective cohort study. N Z Med J. 2018;131(1476):24-39. 29879724

[37] Morris M Seguin M Landon S McKee M Nolte E . Exploring the role of leadership in facilitating change to improve cancer survival: An analysis of experiences in seven high income countries in the International Cancer Benchmarking Partnership (ICBP). Int J Health Pol Manag. 2021. 10.34172/ijhpm.2021.84. Online ahead of print. PMC9808244 34380203

[38] Li M Lakdawalla DN Goldman DP . Association between spending and outcomes for patients with cancer. J Clin Oncol. 2020;38(4):323-331. 31804868 10.1200/JCO.19.01451 PMC6994252

[39] Gatta G Trama A Capocaccia R . Variations in cancer survival and patterns of care across Europe: Roles of wealth and health-care organization. J Natl Cancer Inst Monogr. 2013;46:79-87. doi:10.1093/jncimonographs/lgt004 23962511

[40] Philipson T Eber M Lakdawalla DN Corral M Conti R Goldman DP . An analysis of whether higher health care spending in the United States versus Europe is “worth it” in the case of cancer. Heal Aff(Millwood). 2012;31(4):667-675. 10.1377/hlthaff.2011.1298 PMC3829769 22492882

[41] Choi HCW Lam KO Pang HHM Tsang SKC Ngan RKC Lee AWM . Global comparison of cancer outcomes: Standardization and correlation with healthcare expenditures. BMC Publ Health. 2019;19(1):1065-7384. 10.1186/s12889-019-7384-y PMC6686500 31391013

[42] World Health Organization . WHO mortality database. 2022. https://platform.who.int/mortalityhttps://platform.who.int/mortality. Published 2022. Accessed November 21, 2022.

[43] Stevens W Philipson TJ Khan ZM MacEwan JP Linthicum MT Goldman DP . Cancer mortality reductions were greatest among countries where cancer care spending rose the most, 1995-2007. Heal Aff(Millwood). 2015;34(4):562-570. 10.1377/hlthaff.2014.0634 25847637

[44] Brown S Castelli M Hunter DJ , et al. How might healthcare systems influence speed of cancer diagnosis: A narrative review. Soc Sci Med. 2014;116:5656-6363. 10.1016/j.socscimed.2014.06.030 PMC4124238 24980792

[45] Morris M Landon S Reguilon I Butler J McKee M Nolte E . Understanding the link between health systems and cancer survival: A novel methodological approach using a system-level conceptual mode. J Cancer Policy. 2020;25:100233.

[46] Pedersen AF Forbes L Brain K , et al. Negative cancer beliefs, recognition of cancer symptoms and anticipated time to help-seeking: An international cancer benchmarking partnership (ICBP) study. BMC Cancer. 2018;18(1):363-4287. 29609534 10.1186/s12885-018-4287-8 PMC5879768

[47] Forbes LJL Simon AE Warburton F , et al. Differences in cancer awareness and beliefs between Australia, Canada, Denmark, Norway, Sweden and the UK (the International Cancer Benchmarking Partnership): Do they contribute to differences in cancer survival&quest. Br J Cancer. 2013;108(2):292-300. 23370208 10.1038/bjc.2012.542 PMC3566814

[48] Seguin M Morris M McKee M Nolte E . There’s not enough bodies to do the demand:” An exploration of key stakeholder views on the role of health service capacity in shaping cancer outcomes in 7 international cancer benchmarking partnership countries. Int J Heal Policy Manag. 2020. doi:10.34172/ijhpm.2020.254. Online ahead of print. PMC9808162 33589567

[49] Htun HW Elwood JM Ioannides S Fishman T Lawrenson R . Investigations and referral for suspected cancer in primary care in New Zealand - a survey linked to the International Cancer Benchmarking Partnership. Eur J Cancer Care. 2017;26:e12634. 10.1111/ecc.12634 28105767

[50] Abel GA Mendonca SC McPhail S Zhou Y Elliss-Brookes L Lyratzopoulos G . Emergency diagnosis of cancer and previous general practice consultations: Insights from linked patient survey data. Br J Gen Pract. 2017;67(659):e377-e387. 28438775 10.3399/bjgp17X690869 PMC5442953

[51] McPhail S Swann R Johnson SA , et al. Risk factors and prognostic implications of diagnosis of cancer within 30 days after an emergency hospital admission (emergency presentation): An International Cancer Benchmarking Partnership (ICBP) population-based study. Lancet Oncol. 2022;23(5):587-600. 35397210 10.1016/S1470-2045(22)00127-9 PMC9046095

[52] Ministry of Health . Health targets: Faster cancer treatment; 2015.

[53] Mathew A . Global survey of clinical oncology workforce. J GlobOncol. 2018;4:1-12. 10.1200/JGO.17.00188 PMC6223442 30241241

[54] Evans J Laking G Strother M , et al. Mind the gap: An analysis of foregone health gains from unfunded cancer medicines in New Zealand. Semin Oncol. 2016;43(6):625-637. 28061980 10.1053/j.seminoncol.2016.10.004

[55] Cheema PK Gavura S Migus M Godman B Yeung L Trudeau ME . International variability in the reimbursement of cancer drugs by publically funded drug programs. Curr Oncol. 2012;19(3):e165-e176. 22670106 10.3747/co.19.946 PMC3364777

[56] Vogler S Vitry A Babar ZU . Cancer drugs in 16 European countries, Australia, and New Zealand: A cross-country price comparison study. Lancet Oncol. 2016;17(1):39-47. 26670089 10.1016/S1470-2045(15)00449-0

[57] Manning J . The herceptin interception: New Zealand’s Pharmac and the herceptin funding issue. N Z L Rev. 2011;2011:663-714.

[58] Cancer Control Agency . Understanding the Gap: An Analysis of the Availability of Cancer Medicines in Aotearoa. Wellington: Te Aho o Te Kahu, Cancer Control Agency; 2022.

[59] OECD . Cancer Care: Assuring Quality to Improve Survival, OECD Health Policy Studies. OECD Publishing; 2013.

